# Evaluation of sleep quality and anxiety in Italian pediatric healthcare workers during the first wave of COVID-19 pandemic

**DOI:** 10.1186/s13104-021-05621-9

**Published:** 2021-06-02

**Authors:** Paola Di Filippo, Marina Attanasi, Giulia Dodi, Annamaria Porreca, Massimiliano Raso, Sabrina Di Pillo, Francesco Chiarelli

**Affiliations:** 1 Department of Pediatrics, University of Chieti, Via dei Vestini 5, 66100 Chieti, Italy; 2 Department of Economic Study, University of Chieti, Chieti, Italy; 3 Center of Excellence on Aging, “G.D’Annunzio” University Foundation, University of Chieti, Chieti, Italy

**Keywords:** Insomnia, Pediatric staff, PSQI, Stress, Anxiety, Zung index, Social support, Self-efficacy, COVID-19, Italy, Female sex

## Abstract

**Objective:**

The aim of this study was to evaluate sleep quality and psychological effects on pediatric healthcare workers during the first wave of COVID-19 epidemic in Italy and to evaluate differences between primary and secondary care operators. Pediatric healthcare workers were involved in an online survey to assess sleep quality, stress and anxiety level, self-efficacy and social support in Italian pediatric healthcare workers during COVID-19 pandemic.

**Results:**

We found that 67.4% of our sample suffered from sleep disturbance and 19.4% of subjects suffered from anxiety. Lower values of anxiety and social support were found in primary care staff compared to secondary care one. The associations between healthcare professional figures (being primary or secondary care operators) and mental health outcomes were not statistically significant. However, sex, age and having a SARS-CoV-2 infected relative/friend had an independent effect on mental health outcomes. It is crucial to provide social and psychological support to pediatric healthcare workers. A tailored psychological screening would be desirable for female healthcare workers and for those who have a SARS-CoV-2 infected relative/friend.

**Supplementary Information:**

The online version contains supplementary material available at 10.1186/s13104-021-05621-9.

## Introduction

The outbreak of coronavirus disease 2019 (COVID-19) caused by SARS-CoV-2 infection in Wuhan City in China, spread quickly around the world [1]. The mental impact on population was huge promoting the development of psychological distress and sleep disturbances [2, 3]. Healthcare workers (HWs) were identified as a population at risk for these psychological issues. They faced enormous pressure, caused by the high infection risk, the fear of spreading the infection to their colleagues and their families, the isolation, the verwork, the difficult patients management and the insufficient personal protective equipment (PPE) [4, 5]. The mental health of adult healthcare staff received widespread attention [6]. However, Chen et al. [7] found that the prevalence of depression and anxiety was significant also among pediatric medical staff.

The aim of this study was to evaluate sleep quality and psychological effects on pediatric HWs during the first wave of COVID-19 pandemic in Italy and to assess differences between pediatric primary and secondary care.

## Main text

### Methods

This cross-sectional study was performed using an online self-administered questionnaire survey. We created the survey using Google form platform and we distributed the link obtained by Whatsapp and Facebook. Each answer was recorded anonymously without the possibility to identify the participants.

The questionnaire (Additional file 1) included 94 questions. It was developed through item generation/reduction as recommended in the guidelines of clinicians’ self-administered surveys [8].

Data were collected from May 15th to May 22nd 2020. We identified 450 pediatric HWs, mostly by sending the link to local chat groups.

Pediatric primary care staff included family pediatricians; in Italy the “*family pediatrician”* is a medical professional who guarantees continuous healthcare assistance along child growth and development. Pediatric secondary care staff included pediatricians, residents and nurses working in some pediatric wards.

Online informed consent was provided by all participants prior to their enrollment and they voluntarily filled out the forms and completed the assessment scales. Choosing a priori the option to not register the incomplete questionnaires in Google Form, only complete questionnaires were included in the statistical analysis.

The questionnaire was composed of several sections (Additional file 1):

- Questions 1–14, aimed to define participants’ socio-demographic characteristics and their expertise in the clinical management of COVID-19 patients; we defined as “*clinical management”* both medical examination and telephone conversation (telemedicine);
- Questions 15–32 explored participants’ sleep quality by using *Pittsburgh Sleep Quality Index* (PSQI) [9], a tool validated for Italian population [10];
- Questions 33–62 evaluated anxiety after traumatic events by using *Stanford Acute Stress Reaction Index* (SASR) [11];
- Questions 63–72 measured anxiety levels by using *Zung Self-Rating Anxiety Score* (Zung index) [12];
- Questions 73–82 assessed participants’ feelings of self-efficacy by using *General Self-Efficacy Scale* (GSES). Self-efficacy is an important factor to achieve recovery from a stressful event [13, 14]; higher scores indicate higher self-efficac [15, 16];
- Questions 83–94 measured participants’ social support by using *Multidimensional Scale of Perceived Social Support* (PSS),a research tool measuring perceptions of support from Family, Friends and a Significant Other [17]. Higher score indicating greater perceived social support [18].

### Statistical analysis

Data were presented as median and range for continuous variables and counts and percentages for categorical variables. Shapiro Wilk test was used to check for a normal distribution.

Mann Whitney U test for numerical variables and Pearson's Chi-squared test for categorical data were used to compare survey’s answers in Primary Care staff and Secondary Care staff. Post-hoc analysis by Chi-squared residuals was performed according to Beasley and Schumacker [19]. Holm-Bonferroni was used as post-hoc test. Pearson’s correlation was used to evaluate the relationship among the study scores (PSQI, SASR, Zung index, GSES, PSS).

A multivariable linear model analysis was fitted to explore the association between healthcare professional figures (being primary or secondary care operators) and mental health outcomes (PSQI, SASR, SAS, GSES, PSS) adjusting for potential confounding factors. Confounders were selected from literature first [20], and were subsequently tested for their association with both determinant and the outcomes, or a change of unadjusted effect estimates of 10% when added to the univariate model.

The customary 0.05 type I error probability was chosen.

All analyses were run in R 3.6.2 [Language and Environment for Statistical Computing. R Core Team, R Foundation for Statistical Computing, Vienna, Austria, 2019; (https://www.R-project.org/)].

## Results

The questionnaire was completed by 175 (response rate 38%) pediatric HWs [median age 37.0 (55.5–31.0); 76.6% women]: 58 pediatricians, 55 pediatric residents and 15 pediatric nurses working in pediatric wards and 47 family pediatricians. HWs’ sociodemographic characteristics and their expertise in the clinical management of COVID-19 patients during pandemic are shown in Table 1.

**Table 1:** Socio-demographic characteristics and occupational data of the respondents

	All (N = 175)	Primary care staff (N = 47)	Secondary care staff (N = 128)	*p* value
Choose your occupation among the following options?				
Hospital pediatrician	58 (33.1)			
Pediatric Resident	55 (31.4)			
Pediatric Nurse	15 (8.6)			
Family pediatrician	47 (28.9)			
What is your age?				^**¶**^**< 0.001**
	37.0 (55.5–31.0)	60.0 (58.0–63.5)	33.0 (29.0–40.0)	
Are you a male or a female?				**0.027**
Male	41 (23.4)	17 (36.2)	24 (18.7)	
Female	134 (76.6)	30 (63.8)	104 (81.3)	
Do you have any son or daughter?				** < 0.001**
No	97 (55.4)	6 (12.8)	91 (71,1)	
Yes	78 (44.6)	41 (87.2)	37 (28.9)	
In which macro-area of Italy do you work?				NS
North	19 (10.8)	8 (17.0)	11 (8.6)	
Center	141 (80.6)	35 (74.5)	106 (82.8)	
South	12 (6.9)	4 (8.5)	8 (6.3)	
Islands	3 (1.7)	0 (0.0)	3 (2.3)	
Do you have any flatmate older than 60 years?				** < 0.001**
No	132 (75.4)	24 (51.1)	108 (84.4)	
Yes	43 (24.6)	23 (48.9)	20 (15.6)	
Do you have any SARS-CoV-2 infected relative/friend?				NS
No	115 (65.7)	33 (70.2)	82 (64.1)	
Yes	60 (34.3)	14 (29.8)	46 (35.9)	
How many COVID-19 patients did you visit or manage by phone?				NS
0	76 (43.4)	17 (36.2)	59 (46.1)	
1–5	87 (49.7)	26 (55.3)	61 (47.7)	
6–15	8 (4.6)	4 (8.5)	4 (3.1)	
> 15	4 (2.3)	0 (0.0)	4 (3.1)	
How many patients with suspected COVID-19 symptoms did you visit or manage by phone?				NS
0	9 (5.1)	1 (2.1)	8 (6.2)	
< 10	109 (61.6)	33 (70.2)	76 (59.4)	
10–30	35 (19.8)	12 (25.6)	23 (18.0)	
31–50	11 (6.2)	0 (0.0)	11 (8.6)	
51–100	10 (5.7)	1 (2.1)	9 (7.0)	
> 100	1 (0.6)	0 (0.0)	1 (0.8)	
How do you judge the adequacy of personal protective equipment in your workplace?				** < 0.001**
Absent	19 (10.9)	17 (36.2)	2 (1.6)	*Abs-exc 0.001 *Abs-poor < 0,001 *Abs-suff. < 0,001
Poor	87(49.7)	0(0.0)	7(5.5)	*Poor-suff. < 0,001
Sufficient	62 (35.4)	29 (61.7)	58 (45.3)	
Excellent	7 (4.0)	1 (2.1)	61 (47.6)	
Did you perform rhino-pharyngeal swab for SARS-CoV-2?				** < 0.001**
No	79 (45.1)	33 (70.2)	46 (35.9)	
Yes	96 (54.9)	14 (29.8)	82 (64.1)	
If Yes, which was the result?				NS
Negative	95 (98.9)	14 (100)	81 (98.8)	
Positive	1 (1.1)	0 (0.0)	1 (1.2)	
Did you perform serologic test for SARS-CoV-2?				**0.006**
No	128 (73.1)	42 (89.4)	86 (67.2)	
Yes	47 (26.9)	5 (10.6)	42 (32.8)	
If Yes, which was the result?				NS
Negative IgG and IgM	45 (95.8)	4 (80.0)	41 (97.6)	
Negative IgG and positive IgM	1 (2.1)	1 (20.0)	0 (0.0)	
Positive IgG and negative IgM	1 (2.1)	0 (0.0)	1 (2.4)	
Positive IgG and IgM	0 (0.0)	0(0.0)	0(0.0)	

Values are absolute numbers (percentages) for categorical data and median (range) for continuous variables. *N* numbers, *NS* not significant;

Pediatric primary care staff consisted offamily pediatricians; pediatric secondary care staff consisted of hospital pediatricians, residents in Pediatrics and pediatric nurses; *COVID-19* novel coronavirus 19 disease, *SARS-CoV-2* Severe Acute Respiratory Syndrome—Coronavirus–2; *Ig* immunoglobulin

^¶^*p* value from Mann Whitney *U* test

*p* value from Pearson’s Chi squared test

Bold formatting to values where the p-value is < 0.05

*adjusted *p* value from post-hoc test (Bonferroni test) for pairwise Chi-squared test comparisons

Median PSQI value resulted 8.0 (5.0–10.0). Specifically 68.6% (120/175) of participants showed a score higher than 5 indicating sleep disturbance, of whom 46.3% (81/175) had a score between 6 and 10 (average sleep quality), 20.0% (35/175) between 11 and 16 (poor sleep quality) and 2.3% (4/175) greater than 16 (very poor sleep quality). The median SASR score resulted 63.0 (39.0–83.0). Median Zung index value resulted 34.0 (30.0–44.0); specifically 19.4% of participants had a score higher than 50 indicating anxiety. Median GSES score resulted 29.0 (25.0–34.0) and median PSS score was 5.9 (5.3–6.5). Data are shown in Table 2.

**Table 2:** Evaluation of the psychological effects on the pediatric health-care workers caused by COVID-19 pandemic in Italy

	All (N = 175)	Primary care staff (N = 47)	Secondary care staff (N = 128)	*p* value
PSQI	8.0 (5.0–10.0)	6.0 (5.0–9.0)	8.0 (5.0–10.0)	*NS*
SASR	63.0 (39.0–83.0)	63.0 (33.0–89.0)	64.0 (41.0–81.2)	*NS*
Zung Index	34.0 (30.0–44.0)	31.0 (29.0–41.5)	35.0 (31.0–44.2)	**0.027**
GSES	29.0 (25.0–34.0)	30.0 (27.0–35.0)	29.0 (24.0–33.0)	*NS*
PSS	5.9 (5.3–6.5)	5.8 (5.2–6.0)	6.0 (5.4–6.6)	** < 0.001**
*Acute post-traumatic stress disorder				^*#*^*NS*
No	82 (47.0)	23(49.9)	59 (46.1)	
Yes	93 (53.0)	24 (51.1)	69 (53.9)	

Values are absolute numbers (percentages) for categorical variables and median (range) for continuous variables. *N* numbers, *NS* not significant; primary care staff consisted of family pediatricians; pediatric secondary care staff consisted of hospital pediatricians, residents in Pediatrics and pediatric nurses

*COVID-19* novel coronavirus 19 disease, *PSQI* Pittsburgh Sleep Quality Index, *SASR* Stanford Acute Stress Reaction Index, *GSES* General Self-Efficacy Scale, *PSS* Perceived Social Support; Acute post-traumatic stress disorder is defined according to DSM-IV criteria

*p* value from Mann Whitney *U* test for continuous variables

#*p* value from Pearson’s Chi squared test for categorical variables; bold formatting to values where the *p*-value is < 0.05

**Fig. 1:**
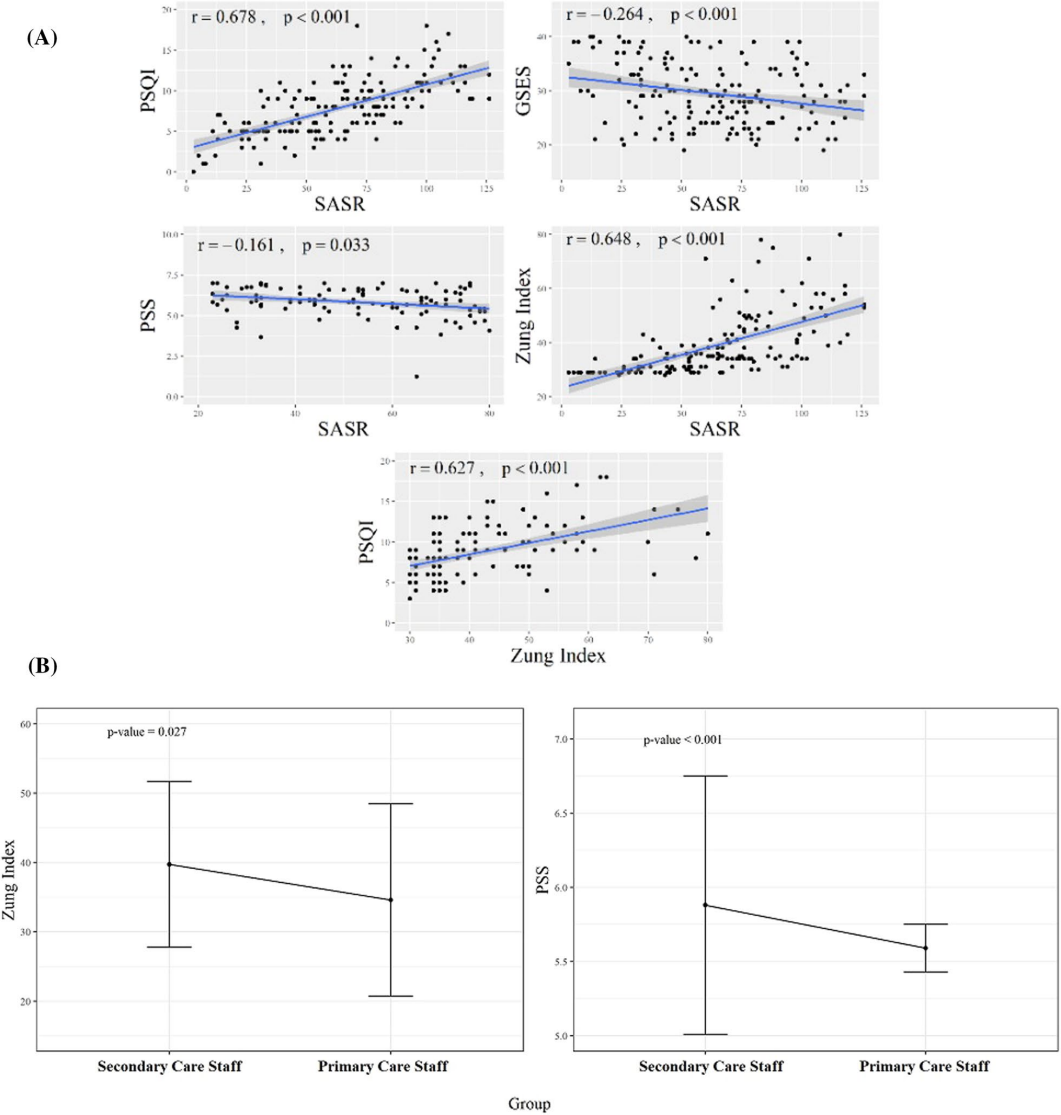
**A** Correlations between the scores used for evaluating COVID-19 pandemic psychological effects on pediatric healthcare workers. *PSQI* Pittsburgh Sleep Quality Index, *SASR* Stanford Acute Stress Reaction Index, *GSES* General Self-Efficacy Scale, *PSS* Perceived Social Support. *p* value was from Pearson correlation. **B** Difference of Anxiety and Social Support between pediatric primary and secondary care staff. *PSS* Perceived Social Support; Values are expressed as median and range. *p* value was from Unpaired *t* test

We found a positive strong correlation between PSQI and SASR (r = 0.678; *p* < 0.001), and between PSQI and Zung index (r = 0.627; *p* < 0.001), and between SASR and Zung index (r = 0.648; *p* < 0.001). Furthermore, a negative weak correlation between SASR and GSES (r = -0.264; *p* < 0.001), and between SASR and PSS (r = -0.161; *p* = 0.033) was found. Data are shown in Fig. 1A.

No significant difference was found for the number of managed COVID-19 patients between primary and secondary care staff. However, the provision of PPE, the percentage of subjects who performed the rhino-pharyngeal swabs and the serology for SARS-CoV-2 were significantly different between the two groups (Table 1).

No differences were found between primary and secondary care staff for PSQI and SASR [6.0 (5.0–9.0) vs. 8.0 (5.0–10.0) *p* = 0.09; 63.0 (33.0–89.0) vs. 64.0 (41.0–81.2) *p* = 0.73, respectively). Lower values of anxiety and social support [31.0 (29.0–41-5) vs. 35.0 (31.0–44.2) *p* = 0.02; 5.8 (5.2–6.0) vs. 6.0 (5.4–6.6) *p* = 0.001 respectively) were found in primary care staff compared to secondary care one (Fig. 1B).

The associations between healthcare professional figures (being primary or secondary care operators) and mental health outcomes (PSQI, SASR, Zung index, GSES, PSS) were not statistically significant even after adjusting for confounders (sex, age, having SARS-CoV-2 infected relative/friend). However, sex had a direct independent effect on PSQI, SASR, Zung index and GSES, age had a direct independent effect on PSS and having at least one SARS-CoV-2 infected relative/friend had a direct independent effect on PSQI, SASR and PSS (Additional file 2).

## Discussion

COVID-19 resulted to be less frequent and severe in children compared to adults [21]. Furthermore, closing schools, lockdown measures and the reluctance to attend pediatric consultations (where the infection risk was high) [22] led to a substantial decrease (ranging from 73 to 88%) in Pediatric Emergency Department visits compared to the same time period in 2019 and 2018 [23, 24]. All these factors created a condition in pediatric wards completely opposed to adult wards which were characterized by running out of beds in a few days [25].

According to these findings, in our study sample we showed a low exposure and infection rate, detected by rhino-pharyngeal swab and serological test (Table 1) compared to all Italian HWs [26].

During the first wave of COVID-19 outbreak we found that 67.4% of participants suffered from sleep disturbance (PSQI > 5) with a median PSQI score of 8.0 (5.0–10.0).

Before COVID-19 pandemic in Italian population the poor sleep quality prevalence (measured by PSQI) was already 40.5% [27] and worsened during lockdown [28]. However, we showed a further increased prevalence of sleep distress in our pediatric staff (67.4%) compared to Italian population (52.4% measured by PSQI) [27]. Our PSQI value resulted higher than the value of Chinese pediatric HWs (7.2 ± 2.62) [29] but lower than Chinese frontline HWs (9.3 ± 3.8) [30].

Furthermore, we found a correlation between stress/anxiety and poor sleep quality confirming their negative effect on sleep of the pediatric HWs during COVID-19 pandemic. On one hand, stress and anxiety are considered the main precipitating factors for insomnia [31, 32]. On the other hand, sleep quality is an important factor regulating behaviors and emotions [33] revealing the bidirectional relationship between sleep quality and psychological distress.

We found a SASR value of 63.0 (39.0–83.0) which was lower than Chinese frontline HWs (77.6 ± 29.5) [34]. Our pediatric staff showed a median Zung index value of 34.0 (30.0–44.0), which was similar to Chinese pediatric HWs (34.4 ± 7.2) [29] and lower than Chinese frontline HWs (55.3 ± 14.2) [34]. Thirty-four participants (19.4%) suffered from anxiety showing a Zung score value ≥ 50. Before COVID-19 pandemic the prevalence of anxiety in Italian population was of 10.3% [35] and increased during the pandemic (21.3%) [25]. In our study sample the prevalence of anxiety was lower compared to Italian population and to frontline HWs (20.6%), and similar to second-line HWs (18.1%) [25]. In a recent meta-analysis with 162.639 participants the prevalence of anxiety was similar between HWs and general population (26.0 and 32.0% respectively), and the highest value was found in Italy compared to other countries [36].

Furthermore, in our pediatric staff we showed a high social support, which could explain the decrease of stress levels. A recent survey with 2166 subjects showed that HWs with less social support had more psychological consequences [37] maybe for the limited opportunities to express their emotions [38, 39].

Regarding the subgroup analysis, primary care staff presented a lower value of anxiety compared to secondary care staff one [31.0 (29.0–41.5) vs. 35.0 (31.0–44.2) *p* = 0.027].

We showed that being primary or secondary care staff was not a risk factor for a worsening of the mental health outcomes (PSQI, SASR, Zung index, GSES, PSS). Noteworthy, female sex showed an independent effect on PSQI, SASR and Zung index. Our findings are in line with previous studies, showing that female sex was associated with increased perception of events as more negative and uncontrollable than male sex predisposing to anxiety [39] and poor sleep quality [40, 41], as also found among medical staff during COVID-19 pandemic [25, 36, 42].

We also showed that having at least one SARS-CoV-2 infected relative/friend had an independent effect on PSQI and SASR, in line with other studies [4, 43]. Moreover, age had an independent effect on PSS. In our study the social support was higher in secondary care compared to primary care staff, probably because family pediatricians were older [42] and work alone without any cooperation with other professional figures.

Concluding, during the first wave of pandemic Italian pediatric HWs suffered from psychological disorders, in particular sleep disturbances and anxiety. Our study emphasizes the necessity of supporting pediatric HWs, which is particularly desirable for female HWs and for those who have at least one SARS-CoV-2 infected relative/friend because they seem to be predictors of anxiety and sleep disorders.

However, further observational studies with larger sample are needed to confirm our findings and longitudinal prospective studies are required to define the long-term mental reverberations in pediatric HWs.

## Limitations

Our study is the first Italian survey investigating sleep quality and psychological status in pediatric HWs and evaluating differences between pediatric primary and secondary care operators.

However, there are some limitations to mention. Firstly, it is a self-administered survey and self-selection bias could have led to an overestimation of effect sizes [44]. Furthermore, self-report measures could convey a further systematic bias. The overall response rate was relatively low, limiting results generalizability. However, the response rate was similar to other surveys in literature [45, 46]. Another important limitation is its cross-sectional nature because of the impossibility of assessing the temporal relationship between exposure and outcome. Lastly, although the two groups had a different composition (only medical doctors in primary care staff) and hence not fully comparable, a recent survey with 2166 respondents showed that nurses and medical doctors suffered from equal anxiety symptoms severity [37].

## Supplementary Information

**Additional file 1.** Survey administered to our study sample. The questionnaire was composed of six sections: **A** socio-demographic characterization of respondents and definition of their expertise in the clinical management of COVID-19 patients during the first wave of pandemic; **B** evaluation of the participants’ sleep quality; **C** evaluation of anxiety after traumatic events; **D** measurement of anxiety levels; **E** participants’ feelings of self-efficacy; **F** participants’ perceived social support.

**Additional file 2.** Multivariable Linear Regression Model to evaluate the association between the type of professional figure (pediatric secondary care vs primary care staff) and health mental outcomes.

## Data Availability

The datasets used and analyzed during the current study are available from the corresponding author on reasonable request.

## Abbreviations

SARS-CoV-2: Severe acute respiratory syndrome coronavirus 2
COVID-19: Coronavirus Disease 2019
HWs: Healthcare Workers
PPE: Personal Protective Equipment
PSQI: Pittsburgh Sleep Quality Index
SASR: Stanford Acute Stress Reaction
SAS: Self- Rating Anxiety Score
GSES: General Self-Efficacy Scale
