# Supplementary material for: Evaluation of sleep quality and anxiety in Italian pediatric healthcare workers during the first wave of COVID-19 pandemic
Source: BMC Res Notes. 2021 Jun 2;14:219. doi: 10.1186/s13104-021-05621-9 (PMC8170450; doi:10.1186/s13104-021-05621-9)
Supplement: Supplementary file 1 — Additional file 1. Survey administered to our study sample. The questionnaire was composed of six sections: A socio-demographic characterization of respondents and definition of their expertise in the clinical management of COVID-19 patients during the first wave of pandemic; B evaluation of the participants’ sleep quality; C evaluation of anxiety after traumatic events; D measurement of anxiety levels; E participants’ feelings of self-efficacy; F participants’ perceived social support. [file 13104_2021_5621_MOESM1_ESM.docx]

**Survey about the COVID-19 pandemic effects on the pediatric health-care workers in Italy**

1. **Socio-demographic characteristics**

Please fill in the questionnaire with your personal information and answer the questions

1. What is your age?

2. Are you a male or a female?

3. Do you have any son or daughter?

4. In which macro-area of Italy do you work?

5. Do you have any flatmate older than 60 years?

6. Choose your occupation among the following options: a) hospital pediatrician; b) family pediatrician; c) resident in pediatrics; d) nurse

7. How many patients with confirmed COVID-19 symptoms did you visit or manage by phone? Choose among the following options: a) 0; b) 1-5; c) 6-15; d) > 15

8. How many patients with suspected COVID-19 symptoms did you visit or manage by phone? Choose among the following options: a) 0; b) <10; c)10-30; d) 31-50; e) 51-100; f) >100;

9. How do you judge the adequacy of personal protective equipment (PPE) provided by your workplace? Choose one of the following options: a) absent; b) poor; c) sufficient; d) excellent

10. Did you perform rhino-pharyngeal swab for SARS-CoV-2?

11. If YES, which was the result?

12. Did you perform serologic test for SARS-Cov-2?

13. If YES, which was the result? Choose one of the following options: a) negative Immunoglobulin(Ig)G and positive IgM; b) negative IgG and IgM; c) positive IgG and negative IgM; d) positive IgG and IgM

14. Have you had any SARS-CoV-2 infected relative or friend?

1. **Pittsburgh Sleep Quality Index (PSQI)**

The following questions relate to your usual sleep habits during the past month only. Your answers should indicate the most accurate reply for the majority of days and nights in the past month. Please answer all questions.

During the past month:

1. When have you usually gone to bed?

2. How long (in minutes) has it taken you to fall asleep each night?

3. When have you usually gotten up in the morning?

4. How many hours of actual sleep do you get at night? (This may be different than the number of hours you spend in bed)

5a. During the past month, how often have you had trouble sleeping because you cannot get to sleep within 30 minutes: a) not during the past month; b) less than once a week; c) once or twice a week; d) three or more times a week

5b. During the past month, how often have you had trouble sleeping because you wake up in the middle of the night or early morning: a) not during the past month; b) less than once a week; c) once or twice a week; d) three or more times a week

5c. During the past month, how often have you had trouble sleeping because you have to get up to use the bathroom: a) not during the past month; b) less than once a week; c) once or twice a week; d) three or more times a week

5d. During the past month, how often have you had trouble sleeping because you cannot breathe comfortably: a) not during the past month; b) less than once a week; c) once or twice a week; d) three or more times a week

5e. During the past month, how often have you had trouble sleeping because you cough or snore loudly: a) not during the past month; b) less than once a week; c) once or twice a week; d) three or more times a week

5f. During the past month, how often have you had trouble sleeping because you feel too cold: a) not during the past month; b) less than once a week; c) once or twice a week; d) three or more times a week

5g. During the past month, how often have you had trouble sleeping because you feel too hot: a) not during the past month; b) less than once a week; c) once or twice a week; d) three or more times a week

5h. During the past month, how often have you had trouble sleeping because you have bad dreams: a) not during the past month; b) less than once a week; c) once or twice a week; d) three or more times a week

5i. During the past month, how often have you had trouble sleeping because you have pain: a) not during the past month; b) less than once a week; c) once or twice a week; d) three or more times a week

5j. Please describe how often you have had trouble sleeping because of other reason(s): a) not during the past month; b) less than once a week; c) once or twice a week; d) three or more times a week

6. During the past month, how often have you taken medicine (prescribed or “over the counter”) to help you sleep? a) not during the past month; b) less than once a week; c) once or twice a week; d) three or more times a week

7. During the past month, how often have you had trouble staying awake while driving, eating meals, or engaging in social activity? a) not during the past month; b) less than once a week; c) once or twice a week; d) three or more times a week

8. During the past month, how much of a problem has it been for you to keep up enthusiasm to get things done? a) not during the past month; b) less than once a week; c) once or twice a week; d) three or more times a week

9. During the past month, how would you rate your sleep quality overall? a) very good; b) fairly good; c) fairly bad; d) very bad

1. **Stanford Acute Stress Reaction Questionnaire (SASR)**

Below there is a list of experiences people sometimes have during and after a stressful event. Please read each item carefully and decide how well it describes your experience during and immediately following the flood (during and in the four weeks afterwards).

Refer to the SARS-CoV-2 pandemic in answering the items below.

Use a 0-5-point scale (0 = not experienced; 1= very rarely experienced; 2= rarely experienced; 3= sometimes experienced; 4= often experienced; 5= very often experienced) and choose the number that best describes your experience.

1. I had difficulty falling or staying asleep: 0; 1; 2; 3; 4; 5
2. I felt restless: 0; 1; 2; 3; 4; 5
3. I felt a sense of timelessness: 0; 1; 2; 3; 4; 5
4. I was slow to respond: 0; 1; 2; 3; 4; 5
5. I tried to avoid feelings about the flood: 0; 1; 2; 3; 4; 5
6. I had repeated distressing dreams of the flood: 0; 1; 2; 3; 4; 5
7. I felt extremely upset if exposed to events that reminded me of an aspect of the flood: 0; 1; 2; 3; 4; 5
8. I would jump in surprise at the least thing: 0; 1; 2; 3; 4; 5
9. The flood made it difficult for me to perform work or other things I needed to do: 0; 1; 2; 3; 4; 5
10. I did not have the usual sense of who I am: 0; 1; 2; 3; 4; 5
11. I tried to avoid activities that reminded me of the flood: 0; 1; 2; 3; 4; 5
12. I felt hypervigilant or "on edge": 0; 1; 2; 3; 4; 5
13. I experienced myself as though I were a stranger: 0; 1; 2; 3; 4; 5
14. I tried to avoid conversations about the flood: 0; 1; 2; 3; 4; 5
15. I had a bodily reaction when exposed to reminders of the flood: 0; 1; 2; 3; 4; 5
16. I had problems remembering important details about the flood: 0; 1; 2; 3; 4; 5
17. I tried to avoid thoughts about the flood: 0; 1; 2; 3; 4; 5
18. Things I saw looked different to me from how I know they really looked: 0; 1; 2; 3; 4; 5
19. I had repeated and unwanted memories of the flood: 0; 1; 2; 3; 4; 5
20. I felt distant from my own emotions: 0; 1; 2; 3; 4; 5
21. I felt irritable or had outbursts of anger: 0; 1; 2; 3; 4; 5
22. I avoided contact with people who reminded me of the flood: 0; 1; 2; 3; 4; 5
23. I would suddenly act or feel as if the flood was happening again: 0; 1; 2; 3; 4; 5
24. My mind went blank: 0; 1; 2; 3; 4; 5
25. I had amnesia for large periods of the flood: 0; 1; 2; 3; 4; 5
26. The flood caused problems in my relationships with other people: 0; 1; 2; 3; 4; 5
27. I had difficulty concentrating: 0; 1; 2; 3; 4; 5
28. I felt estranged or detached from other people: 0; 1; 2; 3; 4; 5
29. I had a vivid sense that the flood was happening all over again: 0; 1; 2; 3; 4; 5
30. I tried to stay away from places that reminded me of the flood: 0; 1; 2; 3; 4; 5

**D. Zung Self- Rating Anxiety Score (SAS)**

For each item decide if it NEVER applies to you (mark 0), SOMETIMES applies to you (mark 1), HALF THE TIME applies to you (mark 2), FREQUENTLY applies to you (mark 3), ALWAYS applies to you (mark 4).

1. I feel tense, nervous, restless, or agitated: a) 0 ; b) 1; c) 2 ; d) 3; e) 4
2. I feel afraid for no apparent reason: a) 0 ; b) 1; c) 2 ; d) 3; e) 4
3. I worry about bad things that might happen to me or those I care about: a) 0 ; b) 1; c) 2 ; d) 3; e) 4
4. I have difficulty falling asleep, staying asleep or waking up early: a) 0 ; b) 1; c) 2 ; d) 3; e) 4
5. I have difficulty eating too much, too little or digesting my food: a) 0 ; b) 1; c) 2 ; d) 3; e) 4
6. I wish I knew a way to make myself more relaxed: a) 0 ; b) 1; c) 2 ; d) 3; e) 4
7. I have difficulty with my concentration, memory or thinking: a) 0 ; b) 1; c) 2 ; d) 3; e) 4
8. I would say I am anxious much of the time: a) 0 ; b) 1; c) 2 ; d) 3; e) 4
9. From time to time I have experienced a racing heartbeat, cold hands or feet, dry mouth, sweating, tight muscles, difficulty breathing, numbness, frequent urination, or hot/cold flashes: a) 0 ; b) 1; c) 2 ; d) 3; e) 4
10. I wish I could be as relaxed with myself as others seem to be: a) 0 ; b) 1; c) 2 ; d) 3; e) 4

**E. General Self-Efficacy Scale (GSES)**

The following scale was developed to evaluate the coping ability of daily living.

Response Format: 1 = Not at all true; 2 = Hardly true; 3 = Moderately true; 4 = Exactly true. Choose the number that best describes your opinion.

1. I can always manage to solve difficult problems if I try hard enough: a) 1; b) 2; c) 3; d) 4
2. If someone opposes me, I can find the means and ways to get what I want: a) 1; b) 2; c) 3; d) 4
3. It is easy for me to stick to my aims and accomplish my goals: a) 1; b) 2; c) 3; d) 4
4. I am confident that I could deal efficiently with unexpected events: a) 1; b) 2; c) 3; d) 4
5. Thanks to my resourcefulness, I know how to handle unforeseen situations: a) 1; b) 2; c) 3; d) 4
6. I can solve most problems if I invest the necessary effort: a) 1; b) 2; c) 3; d) 4
7. I can remain calm when facing difficulties because I can rely on my coping abilities: a) 1; b) 2; c) 3; d)4
8. When I am confronted with a problem, I can usually find several solutions: a) 1; b) 2; c) 3; d) 4
9. If I am in trouble, I can usually think of a solution: a) 1; b) 2; c) 3; d) 4
10. I can usually handle whatever comes my way: a) 1; b) 2; c) 3; d) 4

**F. Multidimensional Scale of Perceived Social Support (MSPSS)**

We are interested in how you feel about the following statements. Read each statement carefully. Indicate how you feel about each statement.

Choose 1 if you very strongly disagree, choose 2 if you strongly disagree, choose 3 if you mildly disagree, choose 4 if you are neutral, choose 5 if you mildly agree, choose 6 if you strongly agree, choose 7 if you very strongly agree.

1. There is a special person who is around when I am in need: a)1; b)2; c)3; d)4; e)5; f)6; g)7
2. There is a special person with whom I can share my joys and sorrows: a)1; b)2; c)3; d)4; e)5; f)6; g)7
3. My family really tries to help me: a)1; b)2; c)3; d)4; e)5; f)6; g)7
4. I get the emotional help and support I need from my family: a)1; b)2; c)3; d)4; e)5; f)6; g)7
5. I have a special person who is a real source of comfort to me: a)1; b)2; c)3; d)4; e)5; f)6; g)7
6. My friends really try to help me: a)1; b)2; c)3; d)4; e)5; f)6; g)7
7. I can count on my friends when things go wrong: a)1; b)2; c)3; d)4; e)5; f)6; g)7
8. I can talk about my problems with my family: a)1; b)2; c)3; d)4; e)5; f)6; g)7
9. I have friends with whom I can share my joys and sorrows : a)1; b)2; c)3; d)4; e)5; f)6; g)7
10. There is a special person in my life who cares about my feelings: a)1; b)2; c)3; d)4; e)5; f)6; g)7
11. My family is willing to help me make decisions: a)1; b)2; c)3; d)4; e)5; f)6; g)7
12. I can talk about my problems with my friends: a)1; b)2; c)3; d)4; e)5; f)6; g)7
