# Supplementary material for: Evaluation of sleep quality and anxiety in Italian pediatric healthcare workers during the first wave of COVID-19 pandemic
Source: BMC Res Notes. 2021 Jun 2;14:219. doi: 10.1186/s13104-021-05621-9 (PMC8170450; doi:10.1186/s13104-021-05621-9)
Supplement: Supplementary file 2 — Additional file 2. Multivariable Linear Regression Model to evaluate the association between the type of professional figure (pediatric secondary care vs primary care staff) and health mental outcomes. [file 13104_2021_5621_MOESM2_ESM.docx]

**Additional file 2. Multivariable Linear Regression Model to evaluate the association between the type of professional figure (pediatric secondary care vs primary care staff) and health mental outcomes**

| Independent variable | Regression coefficient | Standard Error | p value |
| --- | --- | --- | --- |
|  | **PSQI** |  |  |
| Intercept | 5.25 | 1.196 | **<0.001** |
| Primary care staff vs Secondary care staff | -1.06 | 0.783 | 0.178 |
| Female Sex | 2.80 | 0.608 | **<0.001** |
| Age | 0.03 | 0.026 | 0.268 |
| Having SARS-CoV-2 infected relative/friend | -1.34 | 0.529 | **0.012** |
|  | **SASR** |  |  |
| Intercept | 58.73 | 10.399 | **<0.001** |
| * Primary care staff vs Secondary care staff | 7.21 | 6.803 | 0.290 |
| Female Sex | 21.48 | 5.292 | **<0.001** |
| Age | -0.26 | 0.230 | 0.258 |
| Having SARS-CoV-2 infected relative/friend | -9.24 | 4.600 | **0.046** |
|  | **Zung Index** |  |  |
| Intercept | 33.11 | 4.411 | **<0.001** |
| Primary care staff vs Secondary care staff | -1.28 | 2.885 | 0.657 |
| Female Sex | 8.08 | 2.245 | **<0.001** |
| Age | 0.03 | 0.098 | 0.724 |
| Having SARS-CoV-2 infected relative/friend | -3.27 | 1.951 | 0.096 |
|  | **PSS** |  |  |
| Intercept | 6.41 | 0.321 | **<0.001** |
| Primary care staff vs Secondary care staff | 0.24 | 0.210 | 0.252 |
| Female Sex | 0.08 | 0.163 | 0.610 |
| Age | -0.02 | 0.007 | **0.002** |
| Having SARS-CoV-2 infected relative/friend | 0.60 | 0.142 | **<0.001** |
|  | **GSES** |  |  |
| Intercept | 28.45 | 2.035 | **<0.001** |
| Primary care staff vs Secondary care staff | -0.31 | 1.331 | 0.815 |
| Female sex | -2.76 | 1.036 | **0.009** |
| Age | 0.07 | 0.045 | 0.111 |
| Having SARS-CoV-2 infected relative/friend | 0.69 | 0.900 | 0.445 |

**We considered as exposure the belonging to pediatric secondary care staff or to pediatric primary care staff; we adjusted for potential confounders (sex, age, and having SARS-CoV-2 infected relatives/friends);*

*PSQI: Pittsburgh Sleep Quality Index; SASR: Stanford Acute Stress Reaction Index; GSES: General Self-Efficacy Scale; PSS: Perceived Social Support;*

*bold formatting to values where the p-value is <0.05*
